# Use of suppression subtractive hybridisation to extend our knowledge of genome diversity in *Campylobacter jejuni*

**DOI:** 10.1186/1471-2164-8-110

**Published:** 2007-04-30

**Authors:** Philip J Hepworth, Howard Leatherbarrow, C Anthony Hart, Craig Winstanley

**Affiliations:** 1 Division of Medical Microbiology and Genitourinary Medicine, University of Liverpool, Liverpool L69 3GA, UK; 2 DEFRA Epidemiology Fellowship Unit, Department of Veterinary Clinical Science, Faculty of Veterinary Science, Leahurst, Wirral, UK

## Abstract

**Background:**

Previous studies have sought to identify a link between the distribution of variable genes amongst isolates of *Campylobacter jejuni *and particular host preferences. The genomic sequence data available currently was obtained using only isolates from human or chicken hosts. In order to identify variable genes present in isolates from alternative host species, five subtractions between *C. jejuni *isolates from different sources (rabbit, cattle, wild bird) were carried out, designed to assess genomic variability within and between common multilocus sequence type (MLST) clonal complexes (ST-21, ST-42, ST-45 and ST-61).

**Results:**

The vast majority (97%) of the 195 subtracted sequences identified had a best BLASTX match with a *Campylobacter *protein. However, there was considerable variation within and between the four clonal complexes included in the subtractions. The distributions of eight variable sequences, including four with putative roles in the use of alternative terminal electron acceptors, amongst a panel of *C. jejuni *isolates representing diverse sources and STs, were determined.

**Conclusion:**

There was a clear correlation between clonal complex and the distribution of the metabolic genes. In contrast, there was no evidence to support the hypothesis that the distribution of such genes may be related to host preference. The other variable genes studied were also generally distributed according to MLST type. Thus, we found little evidence for widespread horizontal gene transfer between clonal complexes involving these genes.

## Background

Infection due to *Campylobacter *sp. is one of the major causes of diarrheal disease worldwide and is the most common source of bacterial gastroenteritis [1]. Although transmission of *Campylobacter *occurs mainly through the consumption of livestock, with poultry being the most common source, *C. jejuni *has been isolated from diverse animal, human and environmental sources. Several recent studies have sought to determine clonal prevalences amongst isolates from these diverse sources by applying multilocus sequence typing (MLST) [2-7]. Whilst some MLST clonal complexes, such as the ST-21 complex, are widespread, others, such as the ST-61 and ST-42 complexes, have a more restricted distribution amongst different host animals, including humans [4,7].

The complete genome sequences have been published for the *C. jejuni *strains NCTC11168 [8], RM1221 [9] and *C. jejuni *strain 81–176 [10], including its pTet and pVir plasmids [11]. Genome sequences have also been published for strains of *C. lari*, *C. coli *and *C. upsaliensis *[9] and further genome sequence projects for several other *Campylobacter *strains are ongoing [12]. Although unpublished, the genome of *C. fetus *is also complete. In addition, strain-specific DNA sequences have been identified for *C. jejuni *ATCC43431 [13].

There has been considerable interest in characterising genetic variation between isolates of *C. jejuni *with a view to identifying those genes relevant to severity of disease or host colonisation. Inter-strain variations in loci such as those encoding lipooligosaccharide (LOS) [14], capsule [14] or restriction-modification (RM) systems [15] have already been characterised. Comparative genome analyses using microarrays, based largely upon the strain NCTC11168 genome sequence [16-19], indicate high levels of genome diversity but low levels of genome plasticity in *C. jejuni *[20]. Recently it has been suggested that this kind of approach can help to identify genetic markers predictive of the source of an infection [21].

Since these studies were mostly restricted to those genes present in a single strain, NCTC11168, further identification of such markers would be greatly facilitated by the construction of microarrays containing all potentially variable genes. Genes contributing to plasticity amongst *C. jejuni *populations have been identified from the genomes of strains such as RM1221, 81–176 and ATCC43431 [9,11,13,16]. However, it is not clear whether the widely accessible nucleotide and protein sequence databases are representative of the variable genes that occur in *C. jejuni*.

Suppression subtractive hybridisation (SSH) is a method designed to identify sequences present in one strain (the TESTER) but absent from a reference strain (the DRIVER) [22,23] and has been applied previously to identify genetic differences between two *C. jejuni *human isolates with different colonisation potentials [24]. In this study we used several rounds of SSH between and within *C. jejuni *MLST clonal complexes, using isolates from various sources, in order to gain a better understanding of the genomic variability that remains uncharacterised in populations of *C. jejuni*. Furthermore, we tested the hypothesis that the distribution of variable genes involved in the use of alternative terminal electron acceptors may be related to host preference.

## Results and discussion

### SSH libraries

Our initial choice of restriction enzymes for the digestion of DNAs in the SSH was based on a previous study of *C. jejuni *[24]. A summary of all of the SSH sequences obtained is shown in Table 1. The SSH libraries obtained with *Alu*I/*Dra*I-digested DNAs were dominated by very short DNA sequences (92% were <400 bp). In order to obtain longer sequences we carried out two further subtractions using *Rsa*I-digested DNAs. This reduced the proportion of sequences <400 bp in length. The majority of subtracted sequences from strains 670, 504 and 1967 matched sequences previously found in *C. jejuni *strains RM1221 or 81–176. Thus, in an attempt to enrich for sequences not previously reported, the final two subtractions were carried out with strain 1967 (ST-42 complex) as an additional driver to strain NCTC11168 (ST-21 complex). This proved successful in that the majority of SSH sequences no longer matched strains RM1221 or 81–176. However, the overall proportion of SSH sequences matching previously reported *Campylobacter *sp. was not reduced significantly. The combination of enrichment and the choice of an enzyme giving longer SSH fragments resulted in fewer SSH sequences overall (Table 1).

In total, 195 subtracted sequences were obtained. Details of these are given in Additional File 1. These SSH sequences were all confirmed as absent from the driver strain *C. jejuni *NCTC11168, even though occasionally the best BLASTX match in the database was against this strain. Furthermore, the second driver strain, strain 1967, was PCR-negative for nine of 11 sequences tested from the two dual driver subtractions. Of the 195 SSH sequences obtained only two (1%) had a best BLASTX match with proteins from outside the genus *Campylobacter*. A further three SSH sequences (2%) had no significant BLASTX match. Thus, the vast majority of SSH sequences matched within the genus *Campylobacter*. However, it should be noted that eight SSH sequences (4%), although having a best BLASTX match with a *Campylobacter *sp. protein, shared less than 80% identity with the matching protein. Those SSH sequences matching outside the genus *Campylobacter*, matching *Campylobacter *sequences with <80% identity, or used in distribution analysis are shown in Table 2.

Both the mean and the median % GC content for the 195 subtracted sequences was 29%, with a range of 18–42%. The mean % GC contents varied little between the subtractions (28.9–30.8%). Thus, the average % GC contents for the subtracted sequences was only slightly below the values reported for the genomes of the *C. jejuni *strains NCTC11168 (30.6%) [8] or RM1221 (30.3%) [9].

Of the three subtractions conducted using *Alu*I/*Dra*I-digested DNAs and a single driver strain (NCTC11168), two were intra-clonal complex and one was between clonal complexes. The numbers of subtracted sequences obtained did not vary significantly between these subtractions (Table 1). However, there were some variations in the putative functions of subtracted sequences. Plasmid and bacteriophage-associated sequences accounted for 73% of those sequences differing between the two ST-21 complex strains 670 and NCTC11168, and 16% of those sequences differing between the two ST-21 complex strains 504 and NCTC11168, but only 2% of those sequences differing between the ST-42 complex strain 1967 and strain NCTC11168. The largest group of subtracted sequences for the inter-clonal complex subtraction were those associated with metabolism/biosynthesis (29%). The SSH data suggest that there are both intra- and inter-clonal complex variations in genes associated with LOS, capsule, flagella/motility, membrane/transport and metabolism (Table 1).

It has been demonstrated that SSH is an effective method for analysing genetic differences between related strains. In a previous study using SSH, Agron *et al*. [22] were able to detect most of the 7% of genomic differences between two closely related, fully sequenced strains of *Helicobacter pylori*. Unlike in our study, the authors used four parallel subtractions with different restriction enzymes. However, they further demonstrated that as tester-specific sequences became limiting the proportion of repeat fragments increased [22]. Because of our use of single restriction digestion conditions for each subtraction, we cannot claim the extent of coverage achieved by Agron *et al*. [22], but the increased proportion of repeat sequences obtained with the dual driver subtractions is indicative of a reduction in the overall pool of tester-specific sequences in these subtractions. It is our belief that we have detected a significant proportion of the genetic variation between the strains and that our data indicate that there are very few genes present in *C. jejuni *strains with best BLASTX matches outside the genus rather than with already sequenced genes/proteins from within the genus.

### SSH sequences matching outside the genus *Campylobacter*

The two sequences matching outside the genus *Campylobacter *were a putative transport protein (SSH629-D8) and a putative phosphodiesterase (SSH629-D9) (Table 2). These SSH sequences have been submitted to GenBank (EF076761 and EF076762). The predicted protein sequence derived from SSH629-D8 matched a transport protein from *Rhodopseudomonas palustris*, predicted to be a cytoplasmic component of an ABC-type sugar transport system. The gene encoding the phosphodiesterase enzyme from *Delftia acidovorans *matching SSH629-D9 has been cloned and expressed in *Escherichia coli*. It shares sequence similarity to cyclic AMP (cAMP) phosphodiesterase and cyclic nucleotide phosphodiesterases and exhibited activity on cAMP in vivo [25]. Using dot-blot hybridization, we determined the distribution of these two SSH sequences amongst a panel of *C. jejuni *isolates representative of common MLST clonal complexes and various sources (Table 3). The sequences SSH629-D8 and SSH629-D9 were found only in the ST-45 complex (Tables 3 and 4). It should be noted that SSH629-D8 appears to be part of a pseudogene containing a frame-shift mutation. We used PCR amplification to amplify this region from four of the *C. jejuni *isolates testing positive for SSH629-D8, including strain 629, and confirmed that this mutation is genuinely present and not an artefact of the SSH procedure.

### Genomic islands and mobile elements

The distributions of SSH sequences according to the function of the matching proteins varied considerably between strains, reflecting the divergence amongst those strains chosen (Table 1). The SSH data suggest that the ST-21 wild duck isolate, strain 670, contains at least parts of two of the mobile elements reported previously in *C. jejuni *RM1221 (CMLP1 and CJIE4) [9,26]. CMLP1 is a Mu-like bacteriophage and wasn't identified in any of the other tester strains used in this study. CJIE4 carries sequences similar to a putative prophage encoded by *C. lari *(CLIE1) [9]. Strain 670 alone also carried multiple sequences matching the related pTet or pCC31 plasmids reported previously in *C. jejuni *81–176 and *C. coli *respectively [27,28]. SSH data also indicated that the ST-21 cattle isolate strain 504 carries the prophage-like element CJIE4 (18 matching SSH sequences). However, none of the strain 504 SSH sequences matched plasmid sequences. In fact, other than those matching CJIE4, most of the SSH sequences from strain 504 matched hypothetical proteins of unknown function (Table 1). The SSH sequences from strains 1967, 629 and 961, each representing a different MLST type, contained no matches to the genomic islands of strain RM1221 and very few matches to either bacteriophage or plasmid-related sequences (Table 1).

Using dot-blot hybridization we determined the distribution of the sequence 670-D7 as an indicator of the prevalence of the pTet/pCC31 plasmid amongst our strain panel (Table 1). The sequence was found in three isolates from the ST-21 complex and four isolates from the ST-61 complex.

### Plasticity regions and variable genes

Most of the subtractions identified variations in loci previously reported as variable amongst *C. jejuni *strains. It has been reported that variable genes (absent or highly divergent) in the *Campylobacter *genome map to discrete areas, termed variously plasticity regions, hypervariable regions or plasticity zones [16,17,21,29]. Indeed the genotypes of *C. jejuni *strains have been shown to be inherently unstable, and recombination events occur between unrelated strains both *in vitro *and *in vivo *even in the absence of selective pressure [30]. Pearson *et al*. [16] identified seven hypervariable plasticity regions (PR1-7) in the genome of *C. jejuni *NCTC11168. PR6 contains the capsule biosynthesis locus, flanked by capsule transport genes. Twelve SSH sequences in all matched capsule-related proteins. PR7 contains numerous putative outer membrane proteins but also carries a divergent gene encoding a probable flagellar hook protein [16]. Other motility-related genes, including flagellin and flagellin-glycosylation genes, are carried by PR5, along with LOS biosynthesis genes. SSH sequences matching flagellar hook proteins (FlgE), flagellins, flagellin glycosylation proteins and LOS biosynthesis proteins were all identified amongst the SSH sequences in our study. PR4 includes glycosyltransferases and galactosyltransferases of unknown functions as well as genes involved in the sialylation of LOS. SSH sequences matching proteins with similar putative functions were found with both the strain 961 and strain 1967 subtractions. In addition, the 629 subtracted library included one sequence matching both the NCTC11168 *gmhA *gene (from PR4) and a polysaccharide-related protein (SSH629-21).

RM systems protect bacteria from foreign DNA and may impact on the transfer of genes responsible for virulence or host colonisation. It has also been demonstrated previously that RM genes vary in *Campylobacter *spp. [15] and RM genes were amongst those identified in a previous study using SSH [24]. The first three subtractions in our study all identified RM-related SSH sequences. Ahmed *et al*. [24] also identified subtracted sequences relating to arsenite-metabolising genes as present in strain 81–116 but absent from strain NCTC11168, noting that phenylarsonic compounds have been used in poultry feed and may have contaminated agricultural lands on which poultry litter has been used as manure. We identified an SSH matching the arsenical resistance protein of RM1221 in the subtracted library of strain 629 (SSH629-23), a rabbit isolate. In addition, Ahmed *et al*. [24] identified a putative γ-glutamyl transpeptidase gene in their subtraction. Equivalent genes have been implicated in a role in colonization of the gastric mucosa by *Helicobacter pylori *[31,32]. We also identified SSH sequences matching a γ-glutamyl transpeptidase protein in strain 629. It has been reported that *C. jejuni *81–176 carries a putative gene encoding a serine protease belonging to the autotransporter family [10]. The SSH indicates that this gene is also present in the cattle isolate 1967 (SSH1967-A5 and SSH1967-G6). *C. jejuni *81–176 and isolates 1967 are both members of the ST-42 clonal complex. We determined the distribution of SSH1967-A5 amongst our strain panel. All ST-42 isolates were positive for SSH1967-A5. Of the other isolates, only one ST-45 cattle isolate contained SSH1967-A5 (Tables 3 and 4).

We also determined the distribution of the SSH sequence SSH961-A9, which matches an enzyme from *C. jejuni *subsp. *doylei *(Table 2). With the exception of three isolates, the distribution was also restricted to the clonal complex of the tester strain (ST-61 complex; Tables 3 and 4).

### Metabolic genes

When oxygen levels are low *C. jejuni *has the capacity to utilise a wide range of electron acceptors, including fumarate, nitrate, nitrite, sulfite, trimethylamine-*N*-oxide (TMAO) and dimethyl sulfoxide (DMSO) [33-35]. In strain NCTC11168 PR1 contains genes encoding the transport apparatus for molybdenum, which has a putative role in the reduction of nitrate as an alternative terminal electron acceptor [33]. Just upstream of PR1 lies the gene (Cj0264c) encoding the reductase responsible for reduction of TMAO and DMSO under oxygen limiting conditions [33]. Whilst lacking the Cj0264c gene or its close homologues, *C. jejuni *81–176 carries an alternative DMSO reductase gene cluster (*dmsABC-torD*), and an additional cytochrome C biogenenesis gene cluster (*cytC *locus; cju02-09) [10]. Cytochrome C may be an important link between the menaquinine pool and alternative terminal electron acceptors such as DMSO and TMAO [33]. It has been suggested that the capacity of *Campylobacter *isolates to utilise alternative electron acceptors may contribute to selective advantages in specific ecological niches [16], and that the presence of additional respiratory capabilities may contribute to the efficiency of colonisation of highly pathogenic strains such as *C. jejuni *81–176 [10]. Thus, it is possible that the presence or absence of particular genes or islands contributing to growth in microaerophilic environments may influence host preference. Hofreuter *et al*. (2006) recently provided some evidence for this when demonstrating that a *dmsA *mutant of *C. jejuni *81–176 colonised mice less well than its wild-type equivalent in a mixed infection model.

We identified several strain-variable SSH sequences matching putative enzymes with roles in electron transport using alternative terminal electron acceptors. The SSH library constructed from strain 629 (rabbit isolate) included SSH sequences with best BLASTX matches against genes in the cytochrome C biogenesis cluster of *C. jejuni *81–176 (including SSH629-C10). The SSH library constructed from strain 1967 (cattle isolate) included numerous SSH sequences (including SSH1967-D9) with a best BLASTX match against the *dmsABC *genes of *C. jejuni *81–176. The SSH libraries constructed from strains 1967, 670 and strain 504 each contained SSH sequences (SSH1967-C2, SSH670-B10 and SSH504-C10) matching a pyridine nucleotide disulfide oxidoreductase from *C. jejuni *84-25 belonging to a family of enzymes that can play a role in electron transport. SSH sequences SSH670-B10 and SSH504-C10 were identical to each other.

In order to test the hypothesis that the distribution of such metabolic genes may be related to host preference, we chose to screen a panel of isolates varying in MLST type and source for the presence or absence of four representative sequences (Tables 3 and 4). SSH1967-H9 and SSH1967-C10 were chosen to represent the alternative DMSO reductase gene cluster (*dmsABC-torD*), and the additional cytochrome C biogenenesis gene cluster (*cytC *locus; cju02-09), of *C. jejuni *81–176 respectively.  SSH1967-C2 was chosen to represent the pyridine nucleotide disulfide oxidoreductase described in *C. jejuni *84-25. In addition, the Cj0264c gene of strain NCTC11168, encoding a reductase, was included.

Interestingly, of the 81 *C. jejuni *isolates screened, only two (both ST-42) shared the sequences SSH1967-H9 and SSH1967-C2, whilst a further 13 lacked both sequences, including all of the ST-48 isolates. For most of the clonal complexes (ST-21, ST-61, ST-45 and ST-257) the sequences SSH1967-H9 and SSH1967-C2 were mutually exclusive.

There was a strong correlation between the distribution of the two reductase-related sequences (SSH1967-H9 and 1967-C2) or the cytochrome C biogenesis-related sequence (SSH629-C10) and the MLST clonal complex. It has been reported previously that the *C. jejuni *NCTC11168 ORF Cj0264c, which encodes the sole TMAO and DMSO reductase in this strain [33], was absent or highly divergent in 10 strains amongst a panel of 18 *C. jejuini *strains from diverse sources [16]. Our data indicated that this gene was widespread amongst all clonal complexes in our panel of strains, with the exception of the ST-257 complex (Tables 3 and 4).

In our study, there was no evidence for an association between the presence of a particular gene associated with metabolism using alternative electron acceptors and the source of the isolate, suggesting that the presence or absence of these alternative metabolic genes does not play a significant role in niche preferences. However, it should be noted that our strain panel was dominated by isolates from cattle and human sources. Thus we cannot completely rule out such associations with other host species. In a study of gene expression variations by different variants of *C. jejuni *NCTC11168 it was reported that many of the differences in gene expression were in respiration and metabolism genes [36]. The authors suggested that adaptation to different oxygen tensions may influence colonisation potential. The gene expression profiles compared were those of *C. jejuni *NCTC 11168-GS, the genome-sequenced isolate, and NCTC 11168-O, the original isolate from which NCTC11168-GS was derived. Of the two, isolate NCTC 11168-O is a much better coloniser of chicks and invades tissue culture cells far more efficiently [36]. Under microaerobic and severely oxygen-limited conditions there were marked difference in the expression of genes associated with metabolism and respiration. Although ORF Cj0264c was not amongst those loci expressed differently between the two variants of NCTC 11168, such observations support the notion that rather than the presence or absence of metabolic and respiratory genes per se, variations in expression may be more relevant to niche preference.

## Conclusion

In this study we have demonstrated that by broadening the range of clonal complexes and host sources of *C. jejuni *isolates submitted to genetic interrogation, we did not greatly increase the pool of identified strain-variable genes. This suggests that the current database already contains most of the diversity within this species. However, it should also be noted that genes currently associated with other closely related species, such as *C. coli*, can also make a contribution to the diversity within *C. jejuni*.

It is clear from our subtractions between ST-21 complex isolates that variation in gene content occurs within as well as between clonal complexes. However, our survey of strain-variable sequences, including four associated with genes involved in the use of alternative terminal electron acceptors, indicated a distribution according to clonal complex rather than host source. Thus, we found no evidence that the presence or absence of such genes plays a role in the host preferences of *C. jejuni *strains.

## Methods

### Bacterial strains

The bacterial strains used in this study are listed in Table 3 and were isolated in a previous study [7]. Strains for SSH were chosen to enable comparisons within (ST-21 complex) and between MLST clonal complexes. In addition, isolates from diverse animal host sources were chosen (bird, cattle and rabbit; Table 1). These differed from the sources of previously sequenced strains of *C. jejuni *(human or chicken). A panel of isolates representing common MLST clonal complexes (ST-21, ST-42, ST-45, ST-48, ST-257 and ST-61) and different sources were used to study the distribution of subtracted sequences (Table 3). The bacteria were cultured on blood agar at 37°C under microaerophilic conditions.

### MLST

MLST alleles, STs and clonal complexes were assigned using the *Campylobacter *PubMLST database [37] with sequences submitted for allele designation as appropriate.

### Construction and screening of subtraction libraries

Genomic DNA for SSH was isolated from *C. jejuni *strains 670, 504, 1967, 629, 961 and NCTC11168 as described previously [38]. SSH was carried out using the CLONTECH PCR-Select™ Bacterial Genome Subtraction Kit (Clontech) essentially as recommended by the supplier but with a hybridisation temperature of 58°C. In the first three hybridisations, DNAs from *C. jejuni *strains 670, 504 and 1967 respectively were used as tester and DNA from *C. jejuni *NCTC11168 was used as the driver. All DNAs were digested with *Alu*I and *Dra*I. A further two hybridisations, using *Rsa*I-digested DNA from *C. jejuni *strains 629 and 961 respectively as tester, were carried out with dual *Rsa*I-digested driver DNAs from strains 1967 and NCTC11168. PCR amplicons obtained following SSH were cloned into pGEM-T (Invitrogen). The subtraction libraries of *Alu*I/*Dra*I or *Rsa*I fragments thus constructed were screened by sequencing of plasmid DNA extracted from individual clones using M13 forward and reverse vector primers (Lark Technologies). BLAST searches at the *C. jejuni *NCTC11168 genome project web site [39] were used to determine the presence or absence of sequences in the NCTC11168 genome. Sequences absent from the genome of *C. jejuni *NCTC11168 were further analysed using BLASTN and BLASTX searches of the general database using the NCBI website [40] (last accessed 13^th ^October 2006).

### PCR amplification and dot-blot screening of strains

Oligonucleotide primers (Sigma-Genosys) for PCR amplifications are listed in Table 5 along with the annealing temperatures used. DNA for PCR amplifications and dot-blots was prepared by using the Wizard Genomic DNA Purification kit (Promega). For PCR amplification, typically, 1 μl of this DNA was used directly in 25 μl volumes containing 1.25 U of Taq DNA polymerase (Promega), 1 × TaqMaster (Helena Biosciences), 300 nM each primer, 1 × Taq buffer, 2.5 mM MgCl_2 _and 100 μM nucleotides (dATP, dCTP, dGTP, dTTP). Amplifications were carried out in an Eppendorf MasterCycler thermal cycler for 30 cycles consisting of 95°C (1 min), annealing temperature (1 min) and 72°C (2 min) with an additional extension time at 72°C (10 min) following completion of the 30 cycles.

Dot blot hybridisation of genomic DNA was carried out as described previously [38]. Digoxigenin-11-2'-dUTP (DIG) (Roche)-labelled probes were made by carrying out PCR amplification in the presence of 60 μM DIG using vector or internal primers. Hybridisation and subsequent detection of DIG was carried out following the manufacturer's instructions (Roche).

All SSH sequence distributions were determined by dot-blot analysis, with the exception of 629-C10, for which a combination of PCR screening and Southern blots was used. This was because background hybridisation made the 629-C10 dot-blots difficult to interpret. A probe for *flaA *was used in dot blots to confirm the presence of DNA for each strain tested.

## Authors' contributions

PH carried out most of the laboratory work and participated in data analysis. HL carried out the MLST typing. CAH participated in the design, supervision and coordination of the study. CW participated in the design, supervision and coordination of the study, and drafted the manuscript. All authors read and approved the final manuscript.

## Supplementary Material

Additional File 1 **Supplementary Table 1**. The table presents a summary of all the SSH sequences obtained in this study. GC (%); percentage G+C content; %ID; % protein sequence identity. Click here for file

## Figures and Tables

**Table 1:** Summary of SSH experiments.

	**SSH**				
	**670 v 11168**	**504 v 11168**	**1967 v 11168**	**629 v 2 drivers**	**961 v 2 drivers**
Tester MLST group	ST-21	ST-21	ST-42	ST-45	ST-61
Tester source	duck	cattle	cattle	rabbit	rabbit
SSH restriction enzymes	*Alu*I/*Dra*I	*Alu*I/*Dra*I	*Alu*I/*Dra*I	*Rsa*I	*Rsa*I
					
clones sequenced	75	92	98	98	145
unsubtracted	16	40	24	53	60
%subtracted	79%	57%	76%	46%	59%
repeats	7	9	12	28	64
%repeats	12%	17%	16%	53%	75%
subtracted (SSH) sequences	52	43	62	17	21
**SSH sequence length range**					
<200 bp	21	22	14	0	1
200–400 bp	28	20	37	4	5
400–600 bp	3	1	9	4	2
>600 bp	0	0	2	9	13
**Best BLASTX match by strain**					
RM1221 (chicken)	16	23	8	4	4
81–176 (human)	16	1	39	4	0
11168 (human)	2	2	1	1	0
other *C. jejuni*	11	16	12	5	17
other *Campylobacter *sp.	5	1	1	1	0
other genera	0	0	0	2	0
no significant match	2	0	1	0	0
% *Campylobacter*	96%	100%	98%	88%	100%
*Campylobacter *50–80% ID	2	0	2	0	1
*Campylobacter *<50% ID	0	0	0	1	2
**Best BLASTX by function**					
capsule	0	1	6	3	2
LOS	0	0	6	1	6
flagella/motility	0	2	4	1	4
restriction-modification	1	1	4	0	0
membrane/transport	2	5	7	3	3
metabolism/biosynthesis	1	4	18	4	0
plasmid	24	0	1	0	0
bacteriophage	14	7	0	0	2
hypothetical or unknown	7	23	12	2	1
others	1	0	3	3	3
no significant match	2	0	1	0	0

The number of isolates fitting into each category is presented for each of the five subtractions. ID; protein sequence identity

**Table 2:** Summary of selected SSH sequences

**SSH**	**Length (bp)**	**Best BLASTX match/comments [GenBank accession number]**	**%ID**	**Length (AA)**	***E*-value**
		**Best match with another genus**			
629-D8	1116	extracellular solute-binding protein, family 1/ABC transporter (*Rhodopseudomonas palustris *BisB5) [ABE40377]	38	234	3e-36
629-D9	>502	phosphodiesterase (*Delftia acidovorans*) [AAN52089]	42	107	7e-16
					
		**Best match with *C. jejuni *(<80% identity)**			
670-H5	116	Outer membrane lipoprotein MapA CJE1173 (*C. jejuni *RM1221) [AAW35499]	70	31	4e-4
670-2	365	conserved hypothetical protein CJE0262 (*C. jejuni *RM1221) [AAW34855]	76	75	2e-27
1967-D4	272	flagellar hook subunit protein (*C. jejuni *NCTC 11168) [CAB73715]	60	86	3e-18
1967-F11	369	Putative membrane protein CJE0032 (*C. jejuni *RM1221) [AAW34630]	56	107	6e-20
629-E2	1001	putative periplasmic protein (*C. jejuni *81–176) [EAQ73077]	52	209	1e-36
961-E7	750	capsular polysaccharide biosynthesis protein CJE1602 (*C. jejuni *RM1221) [AAW36035]	29	240	3e-17
961-HG5	160	putative sugar transferase, capsule-related (*C. jejuni *176.83) [CAI39731]	47	51	9e-6
961-D5	385	putative glycosyltransferase, LOS-related (*C. jejuni *ATCC 43446) [AAX33825]	52	126	4e-31
					
		**Metabolism using alternative terminal electron acceptors**			
1967-H9	382	anaerobic dimethyl sulfoxide reductase chain B, DmsB, Cju35 (*C. jejuni *81–176) [ABF83738]	100	109	8e-62
1967-C2	310	probable pyridine nucleotide-disulfide oxidoreductase YkgC (*C. jejuni *84-25) [EAQ95476]	100	102	1e-51
629-C10	1213	cytochrome C biogenesis protein, Cju05 (*C. jejuni *81–176) [ABF61591]; gamma-glutamyl transpeptidase, Cju06 (*C. jejuni *81–176) [ABF61592]	100 100	211 118	e-108 1e-92
					
		**Other SSH sequences used in the distribution analysis**			
1967-A5	364	serine protease, subtilase family (*C. jejuni *81–176) [AEQ72903]	100	120	8e-32
961-A9	678	dTDP-glucose pyrophosphorylase (*C. jejuni *subsp. doylei 269.97) [EAU03120]	96	154	3e-74
670-D7	466	putative nickase, cpp17, plasmid pTet/pCC31 – related (*C. jejuni *HB93-13) [EAQ59637]	99	155	5e-77

SSH sequences matching outside the genus *Campylobacter*, sharing less than 80% identity in BLASTX searches with *Campylobacter *proteins, or used in distribution analysis, were included in the table. A full list of SSH sequences is included in Additional file 1. %ID; % protein sequence identity

**Table 3:** Isolates used in this study and distribution of subtracted sequences.

**Isolate**	**ST**	**CC**	**Source**									
				**1967-H9 (Cju35)**	**1967-C2**	**Cj0264c**	**629-C10 (Cju05)**	**629-D8**	**629-D9**	**1967-A5**	**961-A9**	**670-D7**
670	21	21	bird	-	+	+	-	-	-	-	-	+
504	21	21	rabbit	-	+	+	-	-	-	-	-	-
587	21	21	rabbit	-	+	+	-	-	-	-	-	-
597	262	21	cattle	-	-	+	-	-	-	-	-	+
684	522	21	cattle	-	+	+	-	-	-	-	-	-
690	522	21	rabbit	-	+	+	-	-	-	-	-	-
1208	806	21	cattle	-	+	+	-	-	-	-	-	-
1747	19	21	cattle	-	-	+	-	-	-	-	+	-
752	19	21	cattle	-	-	+	-	-	-	-	+	-
1768	53	21	bird	-	-	-	-	-	-	-	-	-
1777	53	21	rabbit	-	-	-	-	-	-	-	-	-
2463	21	21	sheep	-	+	+	-	-	-	-	-	-
1271	21	21	sheep	-	+	+	-	-	-	-	-	-
2057	21	21	sheep	-	+	+	-	-	-	-	-	-
2493	21	21	cattle	-	+	+	-	-	-	-	-	-
2628	21	21	water	-	+	+	-	-	-	-	-	-
11200	21	21	human	-	+	+	-	-	-	-	-	-
11239	86	21	human	-	-	+	-	-	-	-	-	-
11255	21	21	human	-	+	+	-	-	-	-	-	-
11258	53	21	human	-	+	-	-	-	-	-	-	-
11272	50	21	human	-	-	+	-	-	-	-	-	-
564	21	21	cattle	-	+	+	-	-	-	-	+	-
2332	21	21	cattle	-	+	+	-	-	-	-	-	-
870	21	21	cattle	-	+	+	-	-	-	-	-	+
NCTC11168	43	21	human	-	-	+	-	-	-	-	-	-
1107	21	21	cattle	-	+	+	-	-	-	-	-	-
11236	21	21	human	-	+	+	-	-	-	-	-	-
11569	21	21	human	-	+	+	-	-	-	-	-	-
11602	21	21	human	-	+	+	-	-	-	-	-	-
11203	61	61	human	-	+	+	-	-	-	-	+	+
11232	61	61	human	-	+	+	-	-	-	-	+	+
11250	61	61	human	-	+	+	-	-	-	-	+	-
11426	61	61	human	-	+	+	-	-	-	-	+	-
11678	61	61	human	-	+	+	-	-	-	-	+	-
11682	61	61	human	-	+	+	-	-	-	-	+	+
665	61	61	cattle	-	+	+	-	-	-	-	+	-
1141	61	61	cattle	-	+	+	-	-	-	-	+	-
1231	61	61	cattle	-	+	+	-	-	-	-	+	+
1244	61	61	cattle	-	+	+	-	-	-	-	+	-
549	61	61	cattle	-	+	+	-	-	-	-	+	-
961	61	61	rabbit	-	+	+	-	-	-	-	+	-
463	45	45	badger	+	-	+	+	+	+	-	-	-
1949	45	45	badger	+	-	+	+	+	-	-	-	-
2076	45	45	badger	+	-	-	+	+	+	-	-	-
759	45	45	cattle	+	-	+	-	+	+	-	-	-
954	45	45	cattle	+	-	+	+	+	+	+	-	-
1488	45	45	cattle	+	-	+	-	+	+	-	-	-
2305	45	45	cattle	+	-	-	+	+	+	-	-	-
762	45	45	bird	+	-	+	-	+	+	-	-	-
1202	137	45	bird	+	-	+	+	+	+	-	-	-
2021	233	45	bird	+	-	-	+	+	+	-	-	-
2487	97	45	bird	+	-	-	+	+	+	-	-	-
935	45	45	rabbit	+	-	+	+	+	+	-	-	-
745	45	45	rabbit	+	-	+	+	+	+	-	-	-
629	45	45	rabbit	+	-	+	+	+	+	-	-	-
11395	45	45	human	+	-	+	-	+	+	-	-	-
11404	45	45	human	+	-	+	-	+	+	-	-	-
11380	45	45	human	+	-	+	-	+	+	-	-	-
11390	45	45	human	+	-	+	+	+	+	-	-	-
2299	257	257	cattle	+	-	-	-	-	-	-	-	-
11207	257	257	human	+	-	-	-	-	-	-	-	-
11279	257	257	human	+	-	-	-	-	-	-	-	-
11305	257	257	human	+	-	-	-	-	-	-	-	-
11311	257	257	human	+	-	-	-	-	-	-	-	-
11201	257	257	human	+	-	-	-	-	-	-	-	-
11237	257	257	human	+	-	-	-	-	-	-	-	-
11359	42	42	human	+	+	+	-	-	-	+	-	-
11421	42	42	human	+	-	+	+	-	-	+	-	-
11429	42	42	human	+	-	+	+	-	-	+	-	-
11781	42	42	human	+	-	+	+	-	-	+	-	-
11997	42	42	human	+	-	+	+	-	-	+	-	-
11400	42	42	human	+	-	+	+	-	-	+	-	-
1967	42	42	cattle	+	+	+	-	-	-	+	-	-
1262	42	42	cattle	+	-	+	+	-	-	+	-	-
1765	42	42	cattle	+	-	+	+	-	-	+	-	-
490	42	42	cattle	+	-	+	+	-	-	+	-	-
11204	48	48	human	-	-	+	-	-	-	-	-	-
11265	48	48	human	-	-	+	-	-	-	-	-	-
11303	48	48	human	-	-	+	-	-	-	-	-	-
11407	48	48	human	-	-	+	-	-	-	-	-	-
11579	48	48	human	-	-	+	-	-	-	-	-	-
*C. coli *1387			water	-	-	-	-	-	-	-	-	-

The presence (+) or absence (-) of a subtracted sequence is indicated. ST; MLST sequence type. CC; clonal complex

**Table 4:** Summary of SSH sequence distributions amongst a panel of *C. jejuni *isolates

	**Total**	**1967-H9 (Cju35)**	**1967-C2**	**Cj0264c**	**629-C10 (Cju05)**	**629-D8**	**629-D9**	**1967-A5**	**961-A9**	**670-D7**
**ST Complex**										
ST-21	29	0	21 (72%)	26 (90%)	0	0	0	0	3 (10%)	3 (10%)
ST-61	12	0	12 (100%)	12 (100%)	0	0	0	0	12 (100%)	4 (31%)
ST-45	18	18 (100%)	0	14 (78%)	12 (67%)	18 (100%)	17 (94%)	1 (5%)	0	0
ST-42	10	10 (100%)	2 (20%)	10 (100%)	8 (80%)	0	0	10 (100%)	0	0
ST-48	5	0	0	5 (100%)	0	0	0	0	0	0
ST-257	7	7 (100%)	0	0	0	0	0	0	0	0
										
**Source of isolate**										
human	36	16 (44%)	13 (36%)	29 (81%)	6 (17%)	4 (11%)	4 (11%)	6 (17%)	6 (17%)	3 (8%)
cattle	24	9 (38%)	13 (54%)	22 (92%)	5 (19%)	4 (17%)	4 (17%)	5 (19%)	8 (33%)	3 (12%)
rabbit	8	3 (38%)	4 (50%)	7 (88%)	3 (38%)	3 (38%)	3 (38%)	0	1 (13%)	0
bird	6	4 (67%)	1 (17%)	3 (50%)	3 (50%)	4 (67%)	4 (67%)	0	0	1 (17%)
badger	3	3 (100%)	0	2 (67%)	3 (100%)	3 (100%)	2 (67%)	0	0	0
sheep	3	0	3 (100%)	3 (100%)	0	0	0	0	0	0
water	1	0	1 (100%)	1 (100%)	0	0	0	0	0	0
										
total *C. jejuni*	81	35 (43%)	35 (43%)	67 (83%)	20 (25%)	18 (22%)	17 (21%)	11 (13%)	15 (19%)	7 (8%)
										
*C. coli*	1	0	0	0	0	0	0	0	0	0

The number and percentage of isolates carrying each of the subtracted sequences is shown amongst isolates grouped either by MLST clonal complex or source.

**Table 5:** Oligonucleotide primers used in this study

**Primer**	**Sequence (5' to 3')**	**Amplicon size (bp)**	**A.T. (°C)**	**Target**
1967-H9F	GGCGCACCTCAATTTAAC	157	50	1967-H9 (Cju35)
1967-H9R	TCCATGTTCTTCTCTCAG			[*dmsB*]
				
1967-C2F	GCCTTAGGGATTGCTACA	172	50	1967-C2
1967-C2R	CTTATGCTCCATTTAGGC			
				
264F2	ACTCACGTATCTTGCTTC	859	50	Cj0264c
264R2	ACTCGCTGCATTTTGAAG			
				
629-C10F	CAAGTATTATGGCCGGAA	459	50	629-C10 (Cju05)
629-C10R	ATCCTATAACACTCGCAC			[*cytC *locus]
				
629-D8F	AACTACGGATCCACCTACG	339	50	629-D8
629-D8R	AGCGGGGTGTATTTTGGAG			
				
629-D9F2	TTGAGTAAAACGACAGC	249	50	629-D9
629-D9R	TGGCCTTAGAGCTATGGA			
				
961-A9F	CCAAATCCTCAGGTGTTC	326	55	961-A9
961-A9R	ACGAGGTTTAGATGGTTC			
				
670-D7F	TACTGATAATCCGCATTGC	414	50	670-D7
670-D7R	CTTGCATATTCACCCCTT			
				
FlaA1	GCATTTCGTATTAACACACAAATGGTGC	1700	55	*flaA*
FlaA2	CTGTAGTAATCTTAAAACATTTTG			

A.T.; annealing temperature.
