# Supplementary material for: Use of suppression subtractive hybridisation to extend our knowledge of genome diversity in Campylobacter jejuni
Source: BMC Genomics. 2007 Apr 30;8:110. doi: 10.1186/1471-2164-8-110 (PMC1868759; doi:10.1186/1471-2164-8-110)
Supplement: Additional File 1 — Supplementary Table 1. The table presents a summary of all the SSH sequences obtained in this study. GC (%); percentage G+C content; %ID; % protein sequence identity. [file 1471-2164-8-110-S1.doc]

**Supplementary table 1. Summary of all SSH data.**

**(a) SSH using isolate 670 as tester**

| **SSH** | **Length (bp)** | **GC**  **(%)** | **Best BLASTX match/ comments [GenBank accession number]** | **%ID** | | | **Length (AA)** | ***E*-**  **value** | |
| --- | --- | --- | --- | --- | --- | --- | --- | --- | --- |
|  |  |  |  |  | | |  |  | |
|  |  |  | **Restriction-modification related** |  | | |  |  | |
| 670-E7 | 196 | 21 | restriction / modification enzyme CjeI (*C. jejuni* P37) [AAF77188] | 100 | | | 65 | 5e-30 |  |
|  |  |  |  |  | | |  |  | |
|  |  |  | **Membrane / transport proteins** |  | | |  |  | |
| 670-H5 | 116 | 25 | outer membrane lipoprotein MapA CJE1173 (*C. jejuni* RM1221) [AAW35499] | 70 | | | 31 | 4e-4 | |
| 670-B4 | 184 | 28 | putative molybdate binding lipoprotein ModA Cj0303c (*C. jejuni* NCTC11168) [CAB72770] | 100 | | | 42 | 2e-16 |  |
|  |  |  |  |  | | |  |  | |
|  |  |  | **Metabolism / biosynthesis** |  | | |  |  | |
| 670-B10 | 368 | 27 | pyridine nucleotide disulfide oxidoreductase YkgC (*C. jejuni* 84-25) [EAQ95476] | 100 | | 122 | | 9e-63 |  |
|  |  |  |  |  | | |  |  | |
|  |  |  | **Bacteriophage or plasmid related** |  | | |  |  | |
| 670-F2 | 164 | 23 | putative phage repressor protein CJE0215 (*C. jejuni* RM1221) [AAW34809] | 100 | | | 21 | 0.003 |  |
| 670-E9 | 239 | 28 | DNA adenine methylase (*C. jejuni* CF93-6) [EAQ57799] | 100 | | | 79 | 2e-38 |  |
| 670-E2 | 155 | 30 | putative phage virion morphogenesis protein CJE0221 (*C.jejuni* RM1221) [AAW34815] | 93 | | | 32 | 2e-9 | |
| 670-A10 | 123 | 37 | putative phage major tail tube protein CJE0226 (*C. jejuni* RM1221) [AAW34820] | 100 | | | 40 | 2e-15 | |
| 670-6 | 315 | 30 | hypothetical protein (*C. jejuni* CF93-6) [EAQ57568] | 100 | | | 28 | 2e-8 |  |
| 670-C11 | 328 | 34 | hypothetical protein (*C. jejuni* 260.94) [EAQ58721] | 98 | | | 109 | 1e-58 |  |
| 670-H12 | 129 | 32 | putative baseplate assembly protein V putative CJE0236 (*C. jejuni* RM1221) [AAW34830] | 100 | | | 42 | 2e-17 | |
| 670-G2 | 162 | 24 | putative lipoprotein CJE0239 (*C. jejuni* RM1221) [AAW34833] | 100 | | | 37 | 3e-13 | |
| 670-B2 | 145 | 32 | putative Mu-like prophage 1 protein CJE0244 (*C. jejuni* RM1221) [AAW34837] | 100 | | | 48 | 4e-20 | |
| 670-F3 | 230 | 39 | phage uncharacterised protein CJE0249 (*C. jejuni* RM1221) [AAW34842] | 100 | | | 76 | 9e-38 | |
| 670-G3 | 202 | 36 | putative prophage MuSo1 F protein CJE0251 (*C. jejuni* RM1221) [AAW34844] | 100 | | | 67 | 6e-36 | |
| 670-A9 | 151 | 30 | putative tail protein X CJE0253 (*C. jejuni* RM1221) [AAW34846] | 100 | | | 40 | e-15 | |
| 670-H11 | 233 | 32 | hypothetical protein CJE0258 (*C. jejuni* RM1221) [AAW34851] | 100 | | | 73 | 3e-36 | |
| 670-C5 | 289 | 33 | major capsid protein HK97 family CJE1458 (*C. jejuni* RM1221) [AAW35900] | 98 | | | 96 | 5e-47 | |
| 670-1 | 188 | 30 | cmgB3/4, type IV secretion protein, pTet plasmid (*C. jejuni* 81-176) [AAR29564] | 100 | | | 62 | 6e-29 | |
| 670-B12 | 478 | 31 | cmgB5, type IV secretion protein, pTet plasmid (*C. jejuni* 81-176). [AAR29569] | 98 | | | 83 | 6e-38 |  |
| 670-G5 | 307 | 25 | cmgB9, putative type IV secretion protein, pTet plasmid (*C. jejuni* 81-176) [AAR29573] | 100 | | | 61 | 8e-28 |  |
| 670-B8 | 150 | 25 | cmgD4, type IV secretion protein, pTet plasmid (*C. jejuni* 81-176) [AAR29576] | 100 | | | 49 | 2e-20 |  |
| 670-7 | 301 | 37 | cpp14, putative DNA primase, pTet plasmid (*C. jejuni* 81-176) [AAR29498] | 100 | | | 100 | 3e-52 |  |
| 670-F6 | 244 | 20 | cpp20, pTet plasmid (*C. jejuni* 81-176) [AAR29553] | 100 | | | 81 | 3e-40 |  |
| 670-A2 | 79 | 25 | cpp20, pTet plasmid (*C. jejuni* 81-176) [AAR29553] | 100 | | | 25 | 6e-07 |  |
| 670-3 | 389 | 31 | cpp22, putative DNA primase, pTet plasmid (*C. jejuni* 81-176) [AAR29555] | 90 | | | 129 | 7e-64 | |
| 670-C6 | 421 | 30 | cpp27, pTet plasmid (*C .jejuni* 81-176) [AAR29560] | 100 | | | 86 | 7e-40 |  |
| 670-E10 | 260 | 35 | cpp33, pTet plasmid (*C. jejuni* 81-176) [AAR29566] | 100 | | | 86 | 2e-45 |  |
| 670-A12 | 293 | 25 | cpp44, pTet plasmid (*C. jejuni* 81-176) [AAR29577] | 98 | | | 97 | 2e-50 |  |
| 670-E6 | 338 | 33 | cpp14, pCC31 plasmid (*C. coli*) [AAR29498] | 95 | | | 80 | 8e-29 |  |
| 670-C2 | 301 | 37 | helicase family protein cpp14 (*C. jejuni* 81-176) [AAR29498] | 100 | 100 | | | 3e-52 |  |
| 670-D7 | 466 | 28 | putative nickase, cpp17 (*C. jejuni* HB93-13) [EAQ59637] | 99 | 155 | | | 5e-77 |  |
| 670-C12 | 211 | 22 | putative nickase, cpp17, pCC31 plasmid (*C. coli*) [AAR29501] | 100 | 48 | | | 5e-20 |  |
| 670-A3 | 292 | 21 | hypothetical protein cpp18 (*C. jejuni* 81-176) [AAR29551] | 100 | 81 | | | 3e-39 |  |
| 670-G11 | 221 | 20 | hypothetical protein cpp19 (*C. jejuni* 81-176) [AAR29552] | 100 | 42 | | | 6e-18 |  |
| 670-D8 | 271 | 32 | hypothetical protein cpp25 (*C. jejuni* HB93-13) [EAQ59629] | 100 | 53 | | | 2e-23 |  |
| 670-D5 | 122 | 38 | DNA topoisomerase III cpp49 (*C. jejuni* 81-176) [AAR29581] | 100 | 40 | | | 2e-16 |  |
| 670-G1 | 183 | 35 | DNA topoisomerase III cpp49 (*C. jejuni* HB93-13) [AAR29581] | 98 | 61 | | | 3e-27 |  |
| 670-D9 | 338 | 28 | DNA topoisomerase III cpp49 (*C. jejuni* 81-176) [AAR29581] | 98 | 92 | | | e-36 |  |
| 670-D3 | 278 | 37 | putative base plate assembly protein, pBT9811 plasmid (*C. coli*  ATCC 51729) [CAB94939] | 100 | | | 92 | 5e-44 |  |
| 670-D4 | 296 | 34 | TraH protein (*C. coli* RM2228) [EAL55853] | 100 | | | 72 | e-36 |  |
| 670-F12 | 252 | 27 | hypothetical protein CCOA0011, pCC178 plasmid (*C. coli* RM2228) [EAL55848] | 96 | | | 83 | 3e-40 |  |
|  |  |  |  |  | | |  |  | |
|  |  |  | **Hypothetical proteins of unknown function** |  | | |  |  | |
| 670-2 | 365 | 28 | conserved hypothetical protein CJE0262 (*C. jejuni* RM1221) [AAW34855] | 76 | | | 75 | 2e-27 | |
| 670-D11 | 288 | 28 | hypothetical protein CJE1447 (*C. jejuni* RM1221) [AAW35889] | 100 | | | 95 | 4e-49 | |
| 670-E12 | 172 | 24 | hypothetical protein CJE1450 (*C. jejuni* RM1221) [AAW35892] | 100 | | | 38 | 4e-14 |  |
| 670-A8 | 342 | 26 | hypothetical protein CJE1461 (*C. jejuni* RM1221) [AAW35903] | 100 | | | 113 | 4e-49 | |
| 670-E11 | 164 | 26 | hypothetical protein (*C. jejuni* 84-25) [EAQ94969] | 100 | 54 | | | 6e-24 |  |
| 670-D1 | 128 | 21 | hypothetical protein (*C. jejuni* 84-25) [EAQ94969] | 96 | 32 | | | 1e-10 |  |
| 670-G9 | 115 | 26 | hypothetical protein (*C. jejuni* CF93-6) [EAQ56919] | 100 | 37 | | | 1 e-4 |  |
|  |  |  |  |  | | |  |  | |
|  |  |  | **Others** |  | | |  |  |  |
| 670-4 | 164 | 25 | Cj0464, ATP-dependent DNA helicase (*C. jejuni* NCTC11168) [CAB75102] | 95 | | | 24 | 2e-6 |  |
|  |  |  |  |  | | |  |  |  |
|  |  |  | **No significant homology** |  | | |  |  |  |
| 670-E1 | 227 | 26 | NSH |  | | |  |  |  |
| 670-E4 | 178 | 26 | NSH |  | | |  |  |  |
|  |  |  |  |  | | |  |  |  |

**(b) SSH using isolate 504 as tester**

| **SSH** | **Length (bp)** | **GC**  **(%)** | **Best BLASTX match / comments [GenBank accession number]** | **%ID** | **Length (AA)** | ***E*-value** |
| --- | --- | --- | --- | --- | --- | --- |
|  |  |  |  |  |  |  |
|  |  |  | **Capsule related** |  |  |  |
| 504-D3 | 332 | 32 | capsular polysaccharide biosynthesis protein *cpsN* (*C. coli* RM2228) [EAL57281] | 99 | 110 | 9e-57 |
|  |  |  |  |  |  |  |
|  |  |  | **Flagella / motility** |  |  |  |
| 504-A1 | 237 | 36 | FlgE (*C. jejuni* 81-176) [AAP34261] | 87 | 79 | 2e-35 |
| 504-H9 | 398 | 28 | methyl accepting chemotaxis protein (*C. jejuni* 84-25) [EAQ94369] | 100 | 132 | 1e-70 |
|  |  |  |  |  |  |  |
|  |  |  | **Restriction-modification related** |  |  |  |
| 504-F11 | 114 | 33 | type II restriction-modification enzyme (*C. jejuni* 84-25) [EAQ93964] | 100 | 37 | 9e-15 |
|  |  |  |  |  |  |  |
|  |  |  | **Membrane / transport proteins** |  |  |  |
| 504-F7 | 229 | 28 | antibiotic transport protein putative CJE0175 (*C. jejuni* RM1221) [AAW34770] | 100 | 75 | e-36 |
| 504-H8 | 148 | 41 | major outer membrane protein (*C. jejuni* HB93-13) [EAQ61150] | 97 | 48 | 4e-21 |
| 504-E10 | 92 | 29 | twin-arginine translocation pathway signal (*C. jejuni* 84-25) [EAQ94868] | 100 | 30 | 3e-10 |
| 504-B4 | 253 | 30 | twin-arginine translocation pathway signal (*C. jejuni* 84-25) [EAQ94868] | 98 | 83 | 3e-41 |
| 504-C4 | 186 | 34 | copper translocating P-type ATPase CJE1295 (*C. jejuni* 84-25) [EAQ95005] | 100 | 54 | 8e-24 |
|  |  |  |  |  |  |  |
|  |  |  | **Metabolism / biosynthesis** |  |  |  |
| 504-2 | 429 | 32 | putative 3-oxoacyl-(acyl-carrier-protein) synthase CJE1492 (*C. jejuni* RM1221) [AAW35933] | 100 | 83 | 2e-41 |
| 504-1 | 130 | 28 | DNA polymerase III (*C. jejuni* 84-25) [EAQ94858] | 100 | 43 | 2e-17 |
| 504-F5 | 170 | 32 | DNA polymerase III (*C. jejuni* 84-25) [EAQ94858] | 100 | 56 | 3e-27 |
| 504-C10 | 368 | 27 | pyridine nucleotide disulfide oxidoreductase YkgC (*C. jejuni* 84-25) [EAQ95476] | 100 | 122 | 9e-63 |
|  |  |  |  |  |  |  |
|  |  |  | **Phage or plasmid related** |  |  |  |
| 504-D5 | 176 | 34 | site specific recombinase, phage integrase family CJE1418 (*C. jejuni* RM1221) [AAW35737] | 95 | 23 | 2e-04 |
| 504-B11 | 271 | 30 | site specific recombinase, phage integrase family CJE1418 (*C. jejuni* RM1221) [AAW35737] | 100 | 90 | 3e-44 |
| 504-B6 | 215 | 29 | putative DNA binding protein CJE1419 (*C. jejuni* RM1221) [AAW35738] | 100 | 51 | 9e-22 |
| 504-C5 | 289 | 33 | major capsid protein HK97 family CJE1458 (*C. jejuni* RM1221) [AAW35900] | 98 | 96 | 5e-47 |
| 504-A6 | 269 | 29 | putative phage terminase, large subunit CJE1471 (*C. jejuni* RM1221) [AAW35912] | 100 | 89 | e-44 |
| 504-A3 | 183 | 32 | putative phage terminase, large subunit CJE1471 (*C. jejuni* RM1221) [AAW35912] | 100 | 60 | 2e-28 |
| 504-H1 | 197 | 32 | portal protein, HK97 family CJE1469 (*C. jejuni* RM1221) [AAW35910] | 100 | 45 | 2e-18 |
|  |  |  |  |  |  |  |
|  |  |  | **Hypothetical proteins of unknown function** |  |  |  |
| 504-H4 | 249 | 25 | putative RloG protein CJE1430 (*C. jejuni* RM1221) [AAW35749] | 100 | 83 | 2e-38 |
| 504-H12 | 287 | 28 | hypothetical protein (*C. jejuni* 84-25) [EAQ94059] | 97 | 91 | 2e-42 |
| 504-H3 | 140 | 29 | hypothetical protein CJE1424 (*C. jejuni* 84-25) [EAQ95435] | 96 | 31 | 4e-10 |
| 504-H10 | 86 | 31 | hypothetical protein CJE1424 (*C. jejuni* RM1221) [AAW35743] | 100 | 27 | 2e-08 |
| 504-C12 | 246 | 24 | hypothetical protein CJE1443 (*C. jejuni* 84-25) [EAQ95198] | 100 | 35 | 2e-12 |
| 504-B3 | 128 | 29 | hypothetical protein CJE1447 (*C. jejuni* RM1221) [AAW35889] | 100 | 42 | 5e-7 |
| 504-C11 | 289 | 28 | hypothetical protein CJE1447 (*C .jejuni* RM1221) [AAW35889] | 100 | 95 | 4e-49 |
| 504-E12 | 172 | 24 | hypothetical protein CJE1450 (*C. jejuni* RM1221) [AAW35892] | 100 | 38 | 4e-14 |
| 504-G2 | 291 | 29 | hypothetical protein CJE1452 (*C. jejuni* RM1221) [AAW35894] | 100 | 96 | 4e-48 |
| 504-H11 | 158 | 34 | hypothetical protein CJ1459 (*C. jejuni* RM1221) [AAW35901] | 100 | 52 | 9e-12 |
| 504-A7 | 342 | 27 | hypothetical protein CJE1461 (*C. jejuni* RM1221) [AAW35903] | 100 | 113 | 3e-49 |
| 504-B5 | 88 | 32 | hypothetical protein CJE1461 (*C. jejuni* RM1221) [AAW35903] | 100 | 29 | 9e-09 |
| 504-G3 | 147 | 27 | hypothetical protein CJE1461 (*C. jejuni* RM1221) [AAW35903] | 100 | 48 | 3e-19 |
| 504-E3 | 267 | 36 | hypothetical protein CJE1461 (*C .jejuni* RM1221) [AAW35903] | 100 | 88 | e-23 |
| 504-6 | 154 | 27 | hypothetical protein CJE1461 (*C. jejuni* RM1221) [AAW35903] | 100 | 51 | 2e-10 |
| 504-A4 | 197 | 24 | hypothetical protein CJE1481 (*C. jejuni* RM1221) [AAW35922] | 100 | 65 | e-30 |
| 504-B1 | 258 | 25 | hypothetical protein CJE1463 (*C. jejuni* RM1221) [AAW35905] | 100 | 59 | e-27 |
| 504-G7 | 153 | 37 | hypothetical protein Cj0494 (*C. jejuni* NCTC11168) [CAB75132] | 100 | 23 | e-04 |
| 504-C7 | 190 | 30 | hypothetical protein Cj1341c (*C. jejuni* NCTC11168) [CAB73768] | 88 | 63 | 3e-24 |
| 504-8 | 225 | 30 | hypothetical protein (*C. jejuni* HB93-13) [EAQ60526] | 100 | 74 | 5e-39 |
| 504-D8 | 288 | 26 | KAP family P-loop domain protein (*C. jejuni* 84-25) [EAQ95198] | 100 | 95 | 1e-49 |
| 504-4 | 126 | 22 | conserved hypothetical protein (*C. jejuni* 84-25) [EAQ95055] | 100 | 41 | 1e-14 |
| 504-H5 | 93 | 29 | conserved hypothetical protein (*C. jejuni* 84-25) [EAQ94933] | 100 | 30 | 1e-09 |
|  |  |  |  |  |  |  |
|  |  |  |  |  |  |  |

**(c) SSH using isolate 1967 as tester**

| **SSH** | **Length**  **(bp)** | **GC**  **(%)** | **Best BLASTX match / comments [GenBank accession number]** | **%ID** | **Length**  **(AA)** | ***E-***  **value** |
| --- | --- | --- | --- | --- | --- | --- |
|  |  |  |  |  |  |  |
|  |  |  | **Capsule related** |  |  |  |
| 1967-F9 | 174 | 26 | putative capsular biosynthesis sugar kinase CJE1610 (*C. jejuni* RM1221) [AAW36043] | 100 | 32 | e-11 |
| 1967-G11 | 265 | 31 | GDP-L-fucose synthetase (*C. jejuni* 81-176) [EAQ58329] | 100 | 87 | 2e-44 |
| 1967-G2 | 215 | 22 | putative glycosyltransferase, capsular locus (*C. jejuni* ATCC 43456) [AAR01891] | 100 | 71 | 2e-34 |
| 1967-B7 | 322 | 22 | putative glycosyltransferase, capsule locus (*C. jejuni* ATCC 43456) [AAR01892] | 100 | 106 | 4e-46 |
| 1967-F1 | 298 | 29 | protein of unknown function, capsule locus (*C. jejuni* CCUG 10954) [AAR01911] | 100 | 61 | e-30 |
| 1967-E5 | 256 | 18 | putative glycosyltransferase, capsule locus (*C. jejuni* CCUG 10954) [AAR01915] | 100 | 45 | 3e-19 |
|  |  |  |  |  |  |  |
|  |  |  | **LPS / LOS related** |  |  |  |
| 1967-C10 | 325 | 22 | putative lipopolysaccharide core biosynthesis protein CJE1884 (*C. jejuni* RM1221) [AAW34484] | 100 | 52 | 1e-22 |
| 1967-B2 | 147 | 27 | sialyl transferase, lipooligosaccharide related (*C. jejuni* 81-176) [AAL09368] | 97 | 48 | 8e-23 |
| 1967-A1 | 143 | 33 | CMP-NeuNAc synthetase (*C. jejuni* NCTC81-176) [AAL09372] | 100 | 47 | 6e-20 |
| 1967-Cam7 | 262 | 36 | putative UDP GlcNAc dehydratase/reductase, polysaccharide biosynthesis protein, cj1293 (*C. jejuni* 81-176) [ABF83716] | 98 | 87 | 5e-44 |
| 1967-D11 | 215 | 24 | putative glycosyltransferase, LOS-related (*C. jejuni* ATCC 43449) [AAL06001] | 100 | 71 | e-38 |
| 1967-A9 | 306 | 24 | beta-1,4-N-acetylgalactosaminyltransferase (*C. jejuni* OH4384) [AAF31769] | 100 | 101 | 6e-57 |
|  |  |  |  |  |  |  |
|  |  |  | **Flagella related** |  |  |  |
| 1967-B10 | 209 | 29 | flagellin glycosylation-related protein, Cj1333 (*C. jejuni* 81-176) [AAM76284] | 100 | 43 | 7e-31 |
| 1967-D4 | 272 | 36 | flagellar hook subunit protein, FlgE (*C. jejuni* NCTC 11168) [CAB73715] | 60 | 86 | 3e-18 |
| 1967-Cam5 | 195 | 19 | motility accessory factor, Cj1337 (*C. jejuni* 81-176) [AAM76285] | 100 | 50 | 1e-22 |
| 1967-D8 | >731 | 35 | FlaB flagellin *C. jejuni* [B39228] | 100 | 243 | 6e-98 |
|  |  |  |  |  |  |  |
|  |  |  | **Restriction-modification related** |  |  |  |
| 1967-H11 | 420 | 26 | putative type I restriction-modification system HsdS subunit, Cju17 (*C. jejuni* 81-176) [ABF83711] | 100 | 139 | 8e-67 |
| 1967-E9 | 109 | 32 | putative type I restriction-modification system HsdR subunit, Cju20 (*C. jejuni* 81-176) [ABF83714] | 100 | 23 | 2e-6 |
| 1967-B4 | 454 | 31 | putative type I restriction-modification system HsdR subunit, Cju20 (*C. jejuni* 81-176) [ABF83714] | 100 | 150 | 1e-82 |
| 1967-H7 | 186 | 25 | type II restriction modification enzyme (*C. jejuni* HB93-13) [ZP_01072176] | 100 | 61 | 1e-29 |
|  |  |  |  |  |  |  |
|  |  |  | **Membrane / transport proteins** |  |  |  |
| 1967-C5 | 516 | 20 | putative membrane protein (*C. jejuni* HB93-13) [EAQ59756] | 94 | 171 | 2e-80 |
| 1967-D3 | 230 | 25 | putative integral membrane protein (*C. jejuni* HB93-13) [EAQ59734] | 100 | 76 | 4e-39 |
| 1967-F11 | 369 | 21 | putative membrane protein CJE0032 (*C. jejuni* RM1221) [AAW34630] | 56 | 107 | 6e-20 |
| 1967-D5 | 323 | 32 | outer membrane protein (*C. jejuni* 81-176) [EAQ72155] | 100 | 107 | 3e-57 |
| 1967-H6 | 381 | 38 | xanthine/uracil permease family protein, cj1369 (*C. jejuni* 81-176) [ABF83731] | 100 | 126 | 6e-59 |
| 1967-F5 | 259 | 32 | hypothetical protein, putative adhesive protein (*C. jejuni* 81-176) [EAQ72352] | 100 | 86 | 8e-44 |
| 1967-A2 | 303 | 28 | Dcu family anaerobic dicarboxylate transport protein (*C. jejuni* 81-176) [EAQ71951] | 100 | 47 | 1e-18 |
|  |  |  |  |  |  |  |
|  |  |  | **Metabolism / biosynthesis** |  |  |  |
| 1967-H5 | 367 | 32 | anaerobic dimethylsulfoxide reductase chain A, DmsA, Cju34 (*C. jejuni* 81-176) [ABF83737] | 100 | 122 | e-67 |
| 1967-D6 | 197 | 41 | anaerobic dimethylsulfoxide reductase chain A, DmsA, Cju34 (*C. jejuni* 81-176) [ABF83737] | 100 | 62 | 2e-29 |
| 1967-Cam3 | 259 | 32 | anaerobic dimethylsulfoxide reductase chain A, DmsA, Cju34 (*C. jejuni* 81-176) [ABF83737] | 100 | 85 | 2e-43 |
| 1967-H9 | 382 | 35 | anaerobic dimethyl sulfoxide reductase chain B, DmsB, Cju35 (*C. jejuni* 81-176) [ABF83738] | 100 | 109 | 8e-62 |
| 1967-C9 | 567 | 30 | anaerobic dimethyl sulfoxide reductase anchor subunit C-like protein, DmsC, Cju36 (*C. jejuni* 81-176) [ABF83739] | 100 | 152 | 9e-77 |
| 1967-D9 | 209 | 32 | anaerobic dimethyl sulfoxide reductase anchor subunit C-like protein, DmsC, Cju36 (*C. jejuni* 81-176) [ABF83739] | 100 | 69 | e-35 |
| 1967-G4 | 347 | 32 | anaerobic dimethyl sulfoxide reductase anchor subunit C-like protein, DmsC, Cju36 (*C. jejuni* 81-176) [ABF83739] | 100 | 115 | 3e-63 |
| 1967-F10 | >337 | 39 | putative X-Pro dipeptidyl-peptidase, cju38 (*C. jejuni* 81-176) [ABF83743] | 99 | 111 | 1e-64 |
| 1967-C12 | 216 | 38 | putative X-Pro dipeptidyl-peptidase, cju38 (*C. jejuni* 81-176) [ABF83743] | 100 | 71 | 4e-36 |
| 1967-G8 | 311 | 27 | putative X-Pro dipeptidyl-peptidase, cju38 (*C. jejuni* 81-176) [ABF83743] | 100 | 72 | 1e-34 |
| 1967-C2 | 310 | 29 | probable pyridine nucleotide-disulfide oxidoreductase YkgC (*C. jejuni* 84-25) [EAQ95476] | 100 | 102 | 1e-51 |
| 1967-H1 | 421 | 27 | beta-hydroxyacyl-(acyl-carrier-protein) dehydratase FabZ CJE0322 (*C. jejuni* RM1221) [AAW34912] | 100 | 71 | e-34 |
| 1967-C7 | 278 | 25 | biotin biosynthesis protein BioC CJE0349 (*C. jejuni* RM1221) [AAW34938] | 100 | 71 | 9e-35 |
| 1967-C11 | 231 | 24 | carboxypeptidase CJE0708 (*C. jejuni* RM1221) [AAW35782] | 100 | 31 | 3e-11 |
| 1967-G9 | 328 | 30 | para-aminobenzoate synthase glutamine amidotransferase, component I (*C. jejuni* 81-176) [EAU01774] | 100 | 55 | 2e-24 |
| 1967-C6 | 388 | 26 | hydrogenase nickel insertion protein HypA CJE0730 (*C. jejuni* RM1221) [AAW35761] | 98 | 63 | 3e-29 |
| 1967-B1 | 292 | 25 | hypothetical protein, cju29, similar to acyl dehydratase in *Azotobacter vinelandii* (*C. jejuni* 81-176) [ABF83721] | 100 | 96 | 6e-42 |
| 1967-E6 | 182 | 25 | hypothetical protein, cju29, similar to acyl dehydratase in *Azotobacter vinelandii* (*C. jejuni* 81-176) [ABF83721] | 100 | 53 | 7e-24 |
|  |  |  |  |  |  |  |
|  |  |  | **Phage or plasmid related** |  |  |  |
| 1967-F7 | 324 | 26 | TraN protein homolog of plasmid pCU110 (*C. upsaliensis* RM3195) [EAL52603] | 85 | 34 | 3e-10 |
|  |  |  |  |  |  |  |
|  |  |  | **Secreted protease** |  |  |  |
| 1967-A5 | 364 | 42 | serine protease, subtilase family (*C. jejuni* 81-176) [AEQ72903] | 100 | 120 | 8e-32 |
| 1967-G6 | 229 | 35 | serine protease, subtilase family (*C. jejuni* 81-176) [AEQ72903] | 90 | 62 | 3e-24 |
|  |  |  |  |  |  |  |
|  |  |  | **Haemagglutination / adhesion related** |  |  |  |
| 1967-D2 | 592 | 29 | filamentous haemagglutinin domain protein (*C. jejuni* 81-176) [EAQ73077] | 99 | 197 | 4e-106 |
|  |  |  |  |  |  |  |
|  |  |  | **Hypothetical proteins of unknown function** |  |  |  |
| 1967-Cam2 | 255 | 31 | hypothetical protein (*C. jejuni* 81-176) [AEQ73118] | 100 | 84 | 2e-43 |
| 1967-A12 | 258 | 33 | hypothetical protein (*C. jejuni* 81-176) [AEQ73118] | 100 | 85 | 2e-44 |
| 1967-C4 | 263 | 24 | hypothetical protein (*C. jejuni* 81-176) [AEQ73021] | 100 | 25 | 2e-5 |
| 1967-H4 | 91 | 36 | putative periplasmic protein (*C. jejuni* 81-176) [AEQ73077] | 100 | 29 | 6e-8 |
| 1967-E4 | 195 | 31 | hypothetical protein CJE1386 (*C. jejuni* RM1221) [AAW35706] | 92 | 24 | 3e-6 |
| 1967-E2 | 466 | 25 | hypothetical protein, Cju01 (*C. jejuni* 81-176) [ABF50672] | 100 | 151 | 5e-64 |
| 1967-B12 | 156 | 21 | hypothetical protein, Cju01 (*C. jejuni* 81-176) [ABF50672] | 100 | 52 | 2e-22 |
| 1967-G7 | 538 | 25 | hypothetical protein, Cju32 (*C. jejuni* 81-176) [ABF83733] | 100 | 128 | e-67 |
| 1967-C1 | 190 | 28 | hypothetical protein (*C. jejuni* 81-176) [AEQ72341] | 100 | 63 | 4e-31 |
| 1967-B9 | 563 | 30 | hypothetical protein, cj1298 (*C. jejuni* 81-176) [ABF83719] | 99 | 187 | 1e-95 |
| 1967-G10 | 145 | 39 | conserved hypothetical protein, cj1368 (*C. jejuni* 81-176) [ABF83729] | 100 | 47 | 9e-21 |
| 1967-Cam10 | 665 | 23 | hypothetical protein, cju19 (*C. jejuni* 81-176) [ABF83713] | 100 | 168 | 4e-87 |
|  |  |  |  |  |  |  |
|  |  |  | **No significant homology** |  |  |  |
| 1967-B5 | 160 | 23 | NSH |  |  |  |
|  |  |  |  |  |  |  |

**(d) SSH using isolate 629 as tester**

| **SSH** | **Length (bp)** | **GC**  **(%)** | **Best BLASTX match / comments [GenBank accession number]** | **%ID** | **Length (AA)** | ***E*- value** |
| --- | --- | --- | --- | --- | --- | --- |
|  |  |  | **capsule-related** |  |  |  |
| 629-16 | 372 | 33 | putative sugar epimerase, capsule-related (*C. jejuni* 176.83)[CAI38728];  nucleotidyl-sugar pyranose mutase (*C. jejuni* 176.83) [CAI38727] | 92  96 | 69  53 | 1e-30  2e-22 |
| 629-39 | 710 | 29 | GDP-4-keto-6-deoxy-D-sugar-3,5-epimerase-4-reductase, capsule-related (*C. jejuni* 176.83) [CAI38715] | 97 | 236 | 7e-132 |
| 629-E9 | 372 | 26 | putative Na^+^/H^+^ antiporter, capsule-related (*C. jejuni* 176.83) [CAI38704] | 98 | 124 | 4e-61 |
|  |  |  |  |  |  |  |
|  |  |  | **LPS / LOS-related** |  |  |  |
| 629-21 | >703 | 24 | polysaccharide-related protein (*C. jejuni* NCTC11828) [AAK12964];  phosphoheptose isomerise, Cj1149c, *gmhA* (*C. jejuni* NCTC 11168) [CAB73403] | 100  96 | 204  27 | 4e-121  6e-7 |
|  |  |  | **flagella-related** |  |  |  |
| 629-H9 | 362 | 34 | Cj0887c, flagellar hook-associated protein FlaD (*C. jejuni* NCTC 11168) [CAB73145] | 90 | 120 | 4e-57 |
|  |  |  | **membrane / transport** |  |  |  |
| 629-A7 | 680 | 30 | transporter protein CJE1728(*C. jejuni* RM1221) [AAW36155] | 92 | 151 | e-71 |
| 629-D8 | 1116 | 31 | extracellular solute-binding protein, family 1 / ABC transporter (*Rhodopseudomonas palustris* BisB5) [EAO87263] | 38 | 234 | 3e-36 |
|  |  |  | **metabolism / biosynthesis** |  |  |  |
| 629-18 | >531 | 39 | oxidoreductase, zinc-binding dehydrogenase family (*C. jejuni* 93-13) [EAQ60624] | 93 | 173 | 7e-88 |
| 629-20 | 411 | 30 | cytochrome C biogenesis protein, Cju05 (*C. jejuni* 81-176) [ABF61591] | 100 | 137 | 5e-37 |
| 629-C10 | 1213 | 34 | cytochrome C biogenesis protein, Cju05 (*C. jejuni* 81-176) [ABF61591];  gamma-glutamyl transpeptidase, Cju06 (*C. jejuni* 81-176) [ABF61592] | 100  100 | 211  118 | e-108  1e-92 |
| 629-B2 | 747 | 36 | gamma-glutamyl transpeptidase, Cju06 (*C. jejuni* 81-176) [ABF61592] | 100 | 248 | 8e-139 |
| 629-D9 | >502 | 26 | phosphodiesterase (*Delftia acidovorans*) [AAN52089] | 42 | 107 | 7e-16 |
|  |  |  | **regulatory** |  |  |  |
| 629-G1 | 969 | 27 | transcriptional regulator CJE0272 (*C. jejuni* RM1221)[AAW34865] | 88 | 140 | 1e-65 |
|  |  |  | **hypothetical proteins** |  |  |  |
| 629-E2 | 1001 | 30 | putative periplasmic protein (*C. jejuni* 81-176) [EAQ73077] | 52 | 209 | 1e-36 |
| 629-A2 | 403 | 30 | conserved hypothetical protein (*C. coli* RM2228) [EAL57287] | 94 | 134 | 5e-72 |
|  |  |  | **Others** |  |  |  |
| 629-E8 | 1080 | 32 | adenylosuccinate lyase, CJE1582 (*C. jejuni* RM1221) [AAW36016];  MmgE/PrpD family protein (*C. jejuni* 81-176) [EAQ72685] | 99  95 | 213  143 | 6e-115  6e-73 |
| 629-23 | 340 | 32 | arsenical-resistance protein CJE1733 (*C. jejuni* RM1221) [AAW36159] | 100 | 112 | e-51 |
|  |  |  |  |  |  |  |

**(e) SSH using isolate 961 as tester**

| **SSH** | **length (bp)** | **GC**  **(%)** | **Best BLASTX match / comments [GenBank accession number]** | **%ID** | **length**  **(AA)** | ***E-***  **value** |
| --- | --- | --- | --- | --- | --- | --- |
|  |  |  |  |  |  |  |
|  |  |  | **capsule-related** |  |  |  |
| 961-E7 | 750 | 30 | capsular polysaccharide biosynthesis protein CJE1602 (*C. jejuni* RM1221) [AAW36035] | 29 | 240 | 3e-17 |
| 961-HG5 | 160 | 33 | putative sugar transferase, capsule-related (*C. jejuni* 176.83) [CAI39731] | 47 | 51 | 9e-6 |
|  |  |  | **LOS / LPS-related** |  |  |  |
| 961-D5 | 385 | 25 | putative glycosyltransferase, LOS-related (*C. jejuni* ATCC 43446) [AAX33825] | 52 | 126 | 4e-31 |
| 961-G12 | 852 | 25 | putative two-domain glycosyltransferase, LOS-related (*C. jejuni* GB19) [ABF14376] | 98 | 163 | 1e-131 |
| 961-D8 | 652 | 23 | beta-1,4-N-acetylgalactosaminyltransferase, LOS-related (*C. jejuni* ATCC 43438) [AAK91723] | 100 | 217 | 2e-127 |
| 961-F9 | 624 | 36 | putative sialic acid synthase, LOS-related (*C. jejuni* GB11) [AAR82876] | 98 | 195 | 7e-106 |
| 961-F12 | 381 | 33 | putative sialic acid synthase, LOS-related (*C. jejuni* GB11) [AAR82876] | 99 | 118 | 8e-60 |
| 961-A9 | 678 | 25 | dTDP-glucose pyrophosphorylase (*C. jejuni* subsp. *doylei* 269.97) [EAU03120] | 96 | 154 | 3e-74 |
|  |  |  | **Flagella / motility** |  |  |  |
| 961-A6 | 610 | 27 | motility accessory factor (*C. jejuni* HB93-13) [EAQ60903] | 100 | 183 | 2e-98 |
| 961-B8 | 406 | 31 | motility accessory factor (*C. jejuni* HB93-13) [EAQ60903] | 100 | 135 | 2e-74 |
| 961-A4 | 894 | 28 | methyl-accepting chemotaxis protein (*tlpA*) (*C. jejuni* 84-25) [EAQ94369] | 100 | 275 | 9e-152 |
| 961-F1 | 1143 | 31 | methyl-accepting chemotaxis protein (*tlpA*) (*C. jejuni* 84-25) [EAQ94369] | 93 | 381 | 1e-166 |
|  |  |  | **Membrane / transport** |  |  |  |
| 961-E10 | 546 | 32 | membrane transport protein YdfJ (*C. jejuni* HB93-13) [EAQ60118] | 98 | 181 | 1e-98 |
| 961-G10 | 805 | 25 | putative membrane protein CJE0387 (*C. jejuni* RM1221) [AAW34976] | 98 | 194 | 9e-86 |
| 961-7 | 838 | 34 | twin-arginine translocation pathway signal (*C. jejuni* 84-25) [EAQ94868] | 99 | 249 | 4e-143 |
|  |  |  | **Bacteriophage-related** |  |  |  |
| 961-B5 | 633 | 32 | Tgh84, similar to putative integrase from *Wolinella succinogenes* (*C. jejuni* ATCC43431) [AAS99039];  Tgh128, similar to putative integrase from *Wolinella succinogenes* (*C. jejuni* ATCC43431) [AAS99085] | 100  87 | 105  96 | 3e-53  9e-41 |
| 961-B12 | 808 | 32 | Tgh128, similar to putative integrase from *Wolinella succinogenes* (*C. jejuni* TGH9011) [AAS99085] | 100 | 25 | 4e-6 |
|  |  |  | **Hypothetical** |  |  |  |
| 961-2 | >398 | 31 | Tgh086 (*C. jejuni* ATCC 43431) [AAS99037];  Tgh085 (*C. jejuni* ATCC 43431) [AAS99038] | 94  97 | 52  36 | 3e-22  4e-13 |
|  |  |  | **Others** |  |  |  |
| 961-B9 | >742 | 28 | ferric receptor CfrA CJE0847 (*C. jejuni* RM1221) [AAW35184] | 97 | 140 | 2e-63 |
| 961-1 | 380 | 28 | ferric receptor CfrA (*C. jejuni* 260-94) [EAQ58229] | 97 | 126 | 1e-68 |
| 961-D12 | 322 | 32 | L-serine ammonia-lyase CJE1796 (*C. jejuni* RM1221) [AAW36219] | 99 | 107 | e-48 |
|  |  |  |  |  |  |  |
